# Motor cortical excitability predicts cognitive phenotypes in amyotrophic lateral sclerosis

**DOI:** 10.1038/s41598-021-81612-x

**Published:** 2021-01-26

**Authors:** Smriti Agarwal, Elizabeth Highton-Williamson, Jashelle Caga, James Howells, Thanuja Dharmadasa, José M. Matamala, Yan Ma, Kazumoto Shibuya, John R. Hodges, Rebekah M. Ahmed, Steve Vucic, Matthew C. Kiernan

**Affiliations:** 1 Brain and Mind Centre and Sydney Medical School, University of Sydney, Sydney, Australia; 2 Institute of Clinical Neurosciences, Royal Prince Alfred Hospital, Sydney, NSW Australia; 3 Present Address: Neurology Unit, A5, Box 165, Addenbrooke’s Hospital, Hills Road, Cambridge, CB2 0QQ UK

**Keywords:** Neurology, Motor neuron disease, Amyotrophic lateral sclerosis

## Abstract

Amyotrophic lateral sclerosis (ALS) and frontotemporal dementia (FTD) are well-recognised as an extended disease spectrum. This study hypothesised that cortical hyperexcitability, an early pathophysiological abnormality in ALS, would distinguish cognitive phenotypes, as a surrogate marker of pathological disease burden. 61 patients with ALS, matched for disease duration (pure motor ALS, n = 39; ALS with coexistent FTD, ALS-FTD, n = 12; ALS with cognitive/behavioural abnormalities not meeting FTD criteria, ALS-Cog, n = 10) and 30 age-matched healthy controls. Cognitive function on the Addenbrooke’s cognitive examination (ACE) scale, behavioural function on the motor neuron disease behavior scale (MiND-B) and cortical excitability using transcranial magnetic stimulation (TMS) were documented. Cortical resting motor threshold (RMT), lower threshold indicating hyperexcitability, was lower in ALS-FTD (50.2 ± 6.9) compared to controls (64.3 ± 12.6, p < 0.005), while ALS-Cog (63.3 ± 12.7) and ALS (60.8 ± 13.9, not significant) were similar to controls. Short interval intracortical inhibition (SICI) was reduced across all ALS groups compared to controls, indicating hyperexcitability. On receiver operating characteristic curve analysis, RMT differentiated ALS-FTD from ALS (area under the curve AUC = 0.745, p = 0.011). The present study has identified a distinct pattern of cortical excitability across cognitive phenotypes in ALS. As such, assessment of cortical physiology may provide more precise clinical prognostication in ALS.

## Introduction

The role of cortical hyperexcitability in amyotrophic lateral sclerosis (ALS) is being increasingly recognised as a central pathophysiological mechanism that triggers the neurodegenerative cascade^1,2^. Accumulating evidence suggests that the site of origin in ALS is likely to be the neocortex from where the disease may be propagated via prion-like, glutaminergic excitotoxic mechanisms^1,3^.

Involvement of the motor cortex is a consistent finding in studies of ALS pathology, with involvement of Betz cells as well as local cortical neurons^4,5^. Following the discovery of TAR DNA binding protein 43 (TDP-43) as the pathogenic link between ALS and frontotemporal dementia (FTD)^6^, pathological staging of ALS has been described based on the propagation of TDP-43 pathology, and shows that motor cortex involvement is evident in early stages of the disease^7^. Interestingly, histological patterns of TDP-43 pathology in the motor cortex are similar in ALS and FTD while those in the anterior cingulate cortex show some differences^8^. Further evidence for the central role of the motor cortex in disease pathogenesis comes from recent studies demonstrating evidence of Betz cell morphological changes in ALS and ALS-FTD^9^, and immunological abnormalities in close proximity to these cells in ALS^10^.

Imaging studies in ALS have demonstrated structural changes in the motor cortex^11^ and functional changes in resting state brain networks involving the primary motor cortex^12^. Motor cortical thinning may be more prominent in ALS with cognitive and behavioural symptoms^13^, along with involvement of the frontotemporal cortical areas^11^. Furthermore, abnormalities in the white matter tracts underlying motor cortical areas are also consistently seen in imaging studies of ALS^11,14,15^.

Cortical motor neuronal (CM) hyperexcitability can be captured by transcranial magnetic stimulation (TMS) techniques^2,16^. The resting motor threshold (RMT) reflects the ease with which the motor cortical output cells can be excited and provides an index of Betz cell function, while short interval intracortical inhibition (SICI) and intracortical facilitation (ICF) are indicative of inhibitory interneuronal function^17^. SICI reduction, indicative of motor cortical hyperexcitability, is an early feature of ALS^18^, preceding clinical and neurophysiological evidence of peripheral neurodegeneration^19^. It distinguishes ALS from mimic disorders^20,21^, being a sensitive and specific marker for the diagnosis of ALS^22^ and a key predictor of survival^23^. Additionally, a low resting motor threshold (RMT) and greater intracortical facilitation (ICF), again indicative of a hyperexcitable motor cortex, have also been recognised as early features in ALS^1,2^.

Cognitive and behavioural deficits in ALS are well established as part of the ALS frontotemporal dementia (FTD) continuum^24^. Motor cortical hyperexcitability changes have been demonstrated in FTD, although less prominent than those in ALS^25^, reinforcing the concept that these two disorders lie on different ends of the neurodegenerative spectrum.

While clinical features of motor dysfunction in ALS have been well documented in the context of TMS measures of cortical hyperexcitability^2^, the relationship with cognitive dysfunction remains unexplored.

As such, the present study hypothesised that changes in corticomotorneuronal (CM) function may be predictive cognitive abnormalities in ALS, as a surrogate marker of overall disease burden. Given that the rate of progression in ALS-FTD may be more rapid than pure motor ALS^24,26^, one would expect to find a greater degree of CM hyperexcitability in the former, and an intermediate profile in patients with ALS who develop cognitive/behavioural dysfunction, not meeting criteria for FTD. If so, an endophenotype and characteristic profile may become apparent, that could then be applied in a clinical setting and potentially developed as a biomarker in treatment trials.

## Materials and methods

### Study participants

Consecutive patients with ALS referred to a specialist multidisciplinary neurodegenerative clinical service at ForeFront/Brain and Mind Centre, as part of the NHMRC Sydney Health Partners Advanced Healthcare and Clinical Translation Centre, were screened between January 2014 and October 2017. Patients were included if they had undergone detailed cognitive and behavioural testing and concurrent TMS studies to document motor cortical excitability measures.

Ethical approval was in place as granted by the Human Research Ethics Committees of the South-Eastern Sydney Local Health District and the Western Sydney Local Health District. All methods were performed in accordance with relevant guidelines. All participants/next of kin gave written informed consent for inclusion in the study.

Patients with probable or definite ALS (n = 61), as per the revised El Escorial and Awaji criteria^27,28^ seen during the above period were included in the study. International consensus criteria for cognitive and behavioural dysfunction were used to assign diagnostic classification for patients^29^. Pure motor ALS patients (n = 39) did not have significant cognitive or behavioural features as per these criteria; patients with cognitive and/or behavioural features on the FTD spectrum, but not meeting FTD diagnostic criteria were classified as ALS-Cog (n = 10). All patients with a diagnosis of ALS-FTD (n = 12) satisfied the consensus criteria for ALS-FTD^29^.

Age matched healthy controls were recruited, for measuring normative values of motor cortical excitability measures, from among volunteers at the ForeFront clinic. The control participants did not have a history of neurological disease or significant systemic illness.

### Clinical assessment

Clinical assessment and neurophysiological studies including needle EMG was performed in all patients to establish the diagnosis and clinical classification. The Medical Research Council (MRC) grading system was used to assess limb power for each subject. The following muscle groups were assessed bilaterally: shoulder abduction, elbow flexion, elbow extension, wrist dorsiflexion, finger abduction, thumb abduction, hip flexion, knee extension and ankle dorsiflexion on an ordinal scale of 0–5, yielding a maximum possible MRC sum score (MRCSS) of 90. Upper limb score (MRC UL) out of a maximum of 60, lower limb score (MRC LL) out of 30 and abductor pollicis brevis (APB) score (MRC APB) out of 5 were also assigned.

The degree of upper motor neuron (UMN) dysfunction was assessed using a previously validated UMN score^30^ based on the jaw jerk, facial reflex, upper and lower limb deep tendon reflexes and plantar responses. The UMN score ranged from 0 (no UMN dysfunction) to 16 (severe UMN dysfunction).

Site of disease onset was noted as bulbar onset or limb onset.

The Amyotrophic Lateral Sclerosis Functional Rating Scale-Revised (ALSFRS-R)^31^ was used to define the clinical staging of disease. The maximum possible score on ALSFRS-R was 48, with a smaller score indicating a greater degree of motor disability. Disease progression rate^32,33^ was defined as follows:

$$ {\text{Progression rate}} = \left( {48 - {\text{ALSFRS - R}}} \right)/{\text{disease duration in months}}. $$

### Cognitive and behavioural assessment

Cognitive testing was performed using the Addenbrooke’s Cognitive Examination^34^. Subdomain scores on the ACE included attention/orientation, fluency, memory, language and visuospatial function, yielding a maximum score of 100. Letter fluency, category fluency and Trails Making Test^35^ were documented as additional measures of executive function. In the presence of clinical bulbar dysfunction, corrected fluency measures were used incorporating control times required to articulate or copy, to avoid overestimating fluency deficits in these patients. Behavioural symptoms were assessed using the previously validated Motor Neuron Disease Behaviour (MiND-B) scale^36^ with a maximum possible score of 36, with lower scores indicating greater dysfunction. The following behavioural domains were assessed, based on frequency and severity of symptoms: disinhibition, apathy and stereotypy, which are most commonly affected in FTD spectrum disorders.

### TMS evaluation

Transcranial magnetic stimulation was performed according to the paired pulse threshold tracking protocol described previously^18,49^. Magnetic stimulation of the motor cortex was delivered using a 90-mm circular coil placed on the patient’s scalp. The coil position was adjusted to a suitable point on the vertex from where a stable motor-evoked potential (MEP) was recorded with the smallest TMS current. The MEP response was recorded from the APB muscle of the dominant hand at rest, except in one patient where the dominant cortex was inexcitable, so the contralateral side was studied. The Magnetic stimuli were generated by two magnetic stimulators connected via a BiStim (Magstim Co.), which allowed paired stimuli to be delivered through a single coil.

The resting motor threshold (RMT), defined as the single stimulus intensity required to achieve and maintain a target motor-evoked potential of 0.2 mV (± 20%), was established. Cortical motor neuronal (CM) hyperexcitability can be captured by transcranial magnetic stimulation (TMS) techniques which may be useful when reaching a diagnosis of ALS^54–56^. The International Federation of Clinical Neurophysiology guidelines define RMT as the minimum stimulus intensity (% stimulator output) required to elicit a small MEP response (> 50 μV) in a target muscle in 50% of TMS stimulus trials. With the development of threshold tracking techniques, RMT has been redefined as the minimal stimulus intensity required to elicit and maintain a target MEP response on 0.2 mV (± 20%) in the target muscle^18^.

Impulses were then delivered in pairs; a sub-conditioning impulse followed by a test stimulus. The sub-conditioning stimulus intensity was fixed at 70% of the resting motor threshold. The test stimulus intensity was varied to achieve the target MEP of 0.2 mV and the difference between the test stimulus intensity and RMT was recorded, and expressed as a percentage of RMT^18,37^. The interval between the stimuli, or the interstimulus interval (ISI), was varied as the protocol proceeded.

Short-interval intracortical inhibition (SICI) was defined as the percentage increase in test stimulus intensity required to achieve the target motor-evoked potential of 0.2 mV at ISI’s of 1, 1.5, 2, 2.5, 3, 3.5, 4, 5 and 7 ms, whilst intra cortical facilitation (ICF) was measured at ISIs of 10, 15, 20, 25 and 30 ms^18^. Inhibition (SICI) and facilitation (ICF) were calculated as the increase and decrease in intensity using the following equation, respectively:

$$ {\text{Inhibition or Facilitation}} = \left[ {\left( {{\text{Conditioned test stimulus intensity }}{-}{\text{ RMT}}} \right) \, /{\text{ RMT}}} \right] \, \times 100 $$

Average SICI was calculated as the mean of SICI values recorded at each interstimulus interval from 1 to 7 ms. Peak SICI was the highest SICI value recorded between 1 and 7 ms. Average ICF was calculated as the mean of ICF values recorded at each interstimulus interval from 10 and 30 ms.

Following the paired-pulse threshold tracking protocol, the maximum MEP was recorded after three single stimuli at 150% resting motor threshold intensity. Maximal cortical silent period (CSP), defined as the maximum duration of electrical silence following a motor-evoked potential that interfered with ongoing EMG activity, was recorded while patients performed weak voluntary contraction. Three single stimuli at 150% resting motor threshold intensity were administered with resultant silent period measurements averaged to determine the maximum cortical silent period. The duration of the silent period was measured from motor-evoked potential onset to the return of EMG activity^38^.

Data acquisition and stimulation delivery were controlled by QTRACS software (Institute of Neurology, Queen Square).

Riluzole administration was recorded at the time of the TMS evaluation to account for possible impact on TMS variables^39^. No centrally acting drugs for at least 24 h prior to administration of the TMS protocol. In addition, all patients were studied while at rest and encouraged to remain relaxed. If the study data quality was degraded by patient movement, the protocol was recommenced and the initial data discarded.

### Statistical analysis

Statistical analysis was performed using the statistical package SPSS 24. Shapiro–Wilk test of normality was used to assess the suitability of variables for parametric analyses. One-way analysis of variance (ANOVA) for group comparisons followed by post hoc t tests (corrected for multiple comparisons using Bonferroni corrected p-values) were used for normally distributed variables. Non-parametric comparisons were made using the Kruskal Wallis test followed by post hoc Mann–Whitney U tests (corrected for multiple comparisons, p < 0.01 for significance) for variables that were not normally distributed. Categorical values were compared using the Chi-square tests for group comparisons followed by post hoc Fisher exact tests. 2-sided p-values were obtained and considered significant when < 0.05, unless stated otherwise.

To examine the potential clinical utility of TMS variables in prediction of cognitive function, receiver operating characteristic (ROC) curve analysis was applied for cortical excitability measures that differed between the groups. The aim of the ROC analysis was to identify the most sensitive and specific predictors that distinguished the following groups:

1. ALS versus controls
2. ALS-Cog versus controls
3. ALS-FTD versus controls
4. ALS versus ALS-FTD

## Results

### Clinical characteristics

Baseline clinical features are shown in Table 1. While age distribution was similar between ALS, ALS-Cog and ALS-FTD, in terms of gender distribution, there were more females in the ALS-Cog group compared to the other two (p = 0.043). FVC was lower in ALS-FTD (62.7% ± 16.5) compared to ALS (80.1% ± 16.5, p = 0.01). Bulbar onset ALS was similar between ALS (31.6%) and ALS-FTD (50%), but was higher in ALS-Cog (80%) compared with ALS (p = 0.01). While overall level of motor function measured using the ALSFRS-R score was similar between the groups, there was more impairment in fine motor component of ALSFRS-R in ALS-FTD (8.5 ± 2.6) when compared to ALS (10.8 ± 1.7, p = 0.006). Duration of disease symptoms was not significantly different between the three patient groups.

**Table 1:** Baseline characteristics.

	ALS n = 39	ALS-Cog n = 10	ALS-FTD n = 12	Controls n = 30	p-value	Post hoc test
Age in years	61.6 ± 11.3	65.4 ± 9	65.3 ± 11.6	62.8 ± 9.9	0.523	NA
Gender (%male)	61.5%	20%	66.7%	40%	0.043	ALS-Cog < ALS^a^ ALS-Cog < ALS-FTD^a^
Years of education	11.9 ± 2.2	10.1 ± 2.6	13.2 ± 4.4		0.119	NA
FVC	80.1 ± 16.5	71.9 ± 24.9	62.7 ± 16.5		0.036	ALS > ALS-FTD^b^
Disease duration (months)	23.3 ± 21.5	15.5 ± 8.2	23 ± 12.2		0.502	NA
ALSFRS-R	42.7 ± 3.6	37.9 ± 7.8	39.6 ± 5.4		0.079	NA
ALSFRS-R bulbar	10.3 ± 2	8.4 ± 1.9	9.8 ± 2.6		0.044	NS
ALSFRS-R fine motor	10.8 ± 1.7	10 ± 2.4	8.5 ± 2.6		0.037	ALS > ALS-FTD^b^
ALSFRS-R gross motor	10.4 ± 1.9	9.2 ± 3.4	9.9 ± 2.9		0.809	NA
ALSFRS-R respiratory	11.5 ± 0.9	10.3 ± 2.4	11.2 ± 1.7		0.314	NA
Progression rate	0.37 ± 0.35	1 ± 1.3	0.5 ± 0.5		0.209	NA
Limb onset	68.4%	20%	50%		0.020	ALS-Cog < ALS^a^
MRC sum score	81.7 ± 7.5	82.9 ± 5.5	81 ± 8.7		0.718	NA
MRC UL score	54.6 ± 6.1	55.7 ± 4.1	52.7 ± 7.2		0.728	NA
MRC LL score	27.1 ± 7.3	27.2 ± 1.6	28.3 ± 2.4		0.285	NA
MRC APB score	4.5 ± 0.5	4.7 ± 0.5	4.4 ± 0.5		0.476	NA
UMN score	9.6 ± 4.3	5.7 ± 5.9	8.8 ± 5.6		0.175	NA
Riluzole therapy %	52.6%	44.4%	33.3%		0.496	NA

Table showing clinical features including motor features and functional scores on various subdomains of the Amyotrophic lateral sclerosis functioning rating scale—revised version (ALSFRS-R).

ALS, amyotrophic lateral sclerosis; FTD, frontotemporal dementia; MRC score, Medical Research Council score; UMN score, upper motor neuron score; UL, upper limb; LL, lower limb; FVC, forced vital capacity; NA, not applicable.

^a^0.01 < p < 0.05; ^b^0.001 < p < 0.01; ^c^p < 0.001, NA = not applicable, all mean variables presented as mean ± SD.

### Cognitive and behavioural function

Total ACE score was below the normal cut off (< 88)^34^ in both ALS-Cog (82.3 ± 10.2) and ALS-FTD (74.5 ± 8.6) groups. Total MiND-B^36^ scores also showed impairment in ALS-Cog and ALS-FTD patients. There were significant group differences in all measures of cognition tested, as shown in Table 2. Likewise, behavioural scores on MiND-B and subdomain scores were significantly different between ALS, ALS-Cog and ALS-FTD (p < 0.001 for all measures). On post hoc testing, the results for the ALS-Cog group were intermediate between ALS-Cog and pure motor ALS (Table 2).

**Table 2:** Cognitive and behavioural profile.

	ALS n = 39	ALS-Cog n = 10	ALS-FTD n = 12	p-value	Post hoc test
ACE total score	92.9 ± 6	82.3 ± 10.2	74.5 ± 8.6	< 0.001	ALS > ALS-FTD^c^ ALS > ALS-Cog^b^
ACE attention score	17.4 ± 0.9	16.4 ± 1.8	14.1 ± 3.7	< 0.001	ALS > ALS-FTD^c^
ACE memory score	23.4 ± 2.4	21.9 ± 2.6	19.6 ± 2.7	< 0.001	ALS > ALS-FTD^c^
ACE fluency score	11.9 ± 2.1	7.7 ± 3.2	6 ± 3.9	< 0.001	ALS > ALS-FTD^c^ ALS > ALS-Cog^c^
ACE language score	24.7 ± 2.1	21.4 ± 3.4	21.6 ± 2.5	< 0.001	ALS > ALS-FTD^c^ ALS > ALS-Cog^c^
ACE visuospatial subscore	15.6 ± 0.5	13.9 ± 1.9	13.1 ± 2.2	< 0.001	ALS > ALS-FTD^c^ ALS > ALS-Cog^b^
Letter fluency	15.6 ± 4.8	8.7 ± 3.9	6.6 ± 6.2	< 0.001	ALS > ALS-FTD^c^ ALS > ALS-Cog^b^
Category fluency	20.1 ± 4.8	17.6 ± 6.7	10.1 ± 3.9	< 0.001	ALS > ALS-FTD^c^ ALS-Cog > ALS-FTD^a^
Naming	11.9 ± 0.3	11.1 ± 1.5	9.7 ± 1.8	< 0.001	ALS > ALS-FTD^c^
Trails B-A score	46.4 ± 34.1	117.2 ± 99	108.8 ± 50.1	0.001	ALS > ALS-FTD^b^ ALS > ALS-Cog^b^
MIND-B total score	34.5 ± 1.9	31.7 ± 4.3	22.3 ± 6.8	< 0.001	ALS > ALS-FTD^c^ ALS-Cog > ALS-FTD^b^
Disinhibition score	15.5 ± 1.2	15.2 ± 1.3	12.1 ± 3.5	< 0.001	ALS > ALS-FTD^c^
Apathy score	11.3 ± 0.9	8.8 ± 3.5	5.9 ± 2.6	< 0.001	ALS > ALS-FTD^c^
Stereotypy score	7.7 ± 0.7	7.6 ± 1	4.2 ± 1.8	< 0.001	ALS > ALS-FTD^c^ ALS-Cog > ALS-FTD^b^

Distribution of cognitive and behavioural features across the ALS phenotypes is shown. Cognition was measured on the Addenbrooke’s cognitive examination (ACE) score including subdomain scores and additional executive function measures are shown. Behavioural profile was described on the motor neuron disease behavioural (MiND-B) scale.

^a^0.01 < p < 0.05; ^b^0.001 < p < 0.01; ^c^p < 0.001, NA = not applicable.

### Motor cortical function characteristics

**Figure 1:**
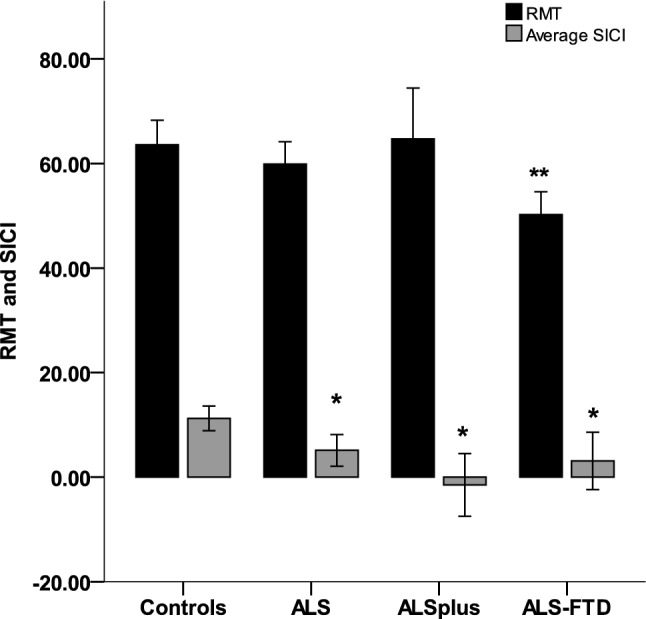
Cortical excitability in ALS. SICI was significantly low in ALS, ALS-Cog and ALS-FTD compared with controls. RMT was low only in ALS-FTD compared with controls. *p < 0.05 compared to controls for all three groups, **p < 0.05 for ALS-FTD compared to controls.

Motor cortical function measures on TMS are summarised in Table 3. Average RMT was lower in ALS-FTD (50.2 ± 6.9) compared with healthy controls (64.3 ± 12.6, p = 0.009). In ALS-Cog and ALS, RMT was similar to control values (Table 3, Fig. 1). Average SICI was lower in all three groups, ALS (5.1 ± 9.2), ALS-Cog (− 1.5 ± 7.8) and ALS-FTD (3.1 ± 8.6) compared with healthy controls (11.3 ± 6, p < 0.001 for group comparisons). Peak SICI was lower in ALS-Cog (8.4 ± 7.6) compared with control values (19.2 ± 8.8, p = 0.019). ICF, CSP and MEP amplitude were similar to control values in all 3 patient groups.

**Table 3:** Motor cortical function on TMS (mean ± SD).

	Controls n = 30	ALS n = 39	ALS-Cog n = 10	ALS-FTD n = 12	p-value	Post hoc test
Resting motor threshold (RMT)	64.3 ± 12.6	60.8 ± 13.9	63.3 ± 12.7	50.2 ± 6.9	0.015	ALS-FTD < HC^b^
Average SICI	11.3 ± 6	5.1 ± 9.2	− 1.5 ± 7.8	3.1 ± 8.6	< 0.001	ALS < HC^a^ ALS-Cog < HC^c^ ALS-FTD < HC^a^
Peak SICI	19.2 ± 8.8	13.6 ± 10.8	8.4 ± 7.6	10.5 ± 6.9	0.005	ALS-Cog < HC^a^
Average ICF	− 3.8 ± 9	− 1.1 ± 7.5	− 2 ± 7.6	0.3 ± 13.5	0.147	NA
CSP	181.7 ± 52.4	186.9 ± 39.9	168.1 ± 38.6	186.1 ± 45.9	0.659	NA
MEP amplitude	2.1 ± 1.9	2.5 ± 1.9	2.7 ± 1.9	3.3 ± 2.1	0.475	NA

Distribution of motor cortical function across the ALS phenotypes is shown.

SICI, short interval intracortical inhibition; ICF, intracortical facilitation; MEP, motor evoked potential; HC, healthy controls.

^a^0.01 < p < 0.05; ^b^ 0.001 < p < 0.01; ^c^ p < 0.001, NA = not applicable.

### ROC analysis

**Figure 2:**
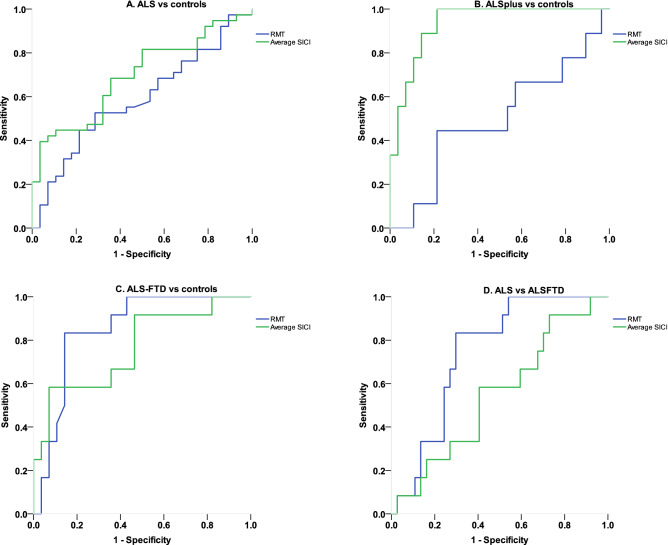
Receiving operating characteristic (ROC) curves for differentiating ALS, ALS-Cog and ALS-FTD from controls. (**A**) Receiver operating characteristic (ROC) curves for differentiating ALS from healthy controls with area under the curve (AUC = 0.586, p = 0.233 for RMT and AUC = 0.695, p = 0.007 for average SICI) (**B**) ROC curves for differentiating ALS-Cog from healthy controls with area under the curve (AUC = 0.500, p = 1 for RMT and AUC = 0.933, p < 0.001 for average SICI) (**C**) ROC curves for differentiating ALS-FTD from healthy controls with area under the curve (AUC = 0.850, p = 0.001 for RMT and AUC = 0.765, p = 0.009 for average SICI) 2D) ROC curves for differentiating ALS from ALS-FTD with area under the curve (AUC = 0.745, p = 0.011 for RMT and AUC = 0.547, p = 0.625 for average SICI).

ROC analysis revealed that average SICI was the most sensitive and specific marker for differentiating ALS and ALS-Cog from healthy controls (Fig. 2A,B). In case of ALS-FTD (Fig. 2C), both RMT and SICI differentiated patients from healthy controls, with RMT being the most sensitive and specific marker. In addition, RMT also was sensitive and specific to differentiate between ALS-FTD and ALS (Fig. 2D, Area under the curve AUC = 0.745, p = 0.011) while SICI (AUC = 0.547, p = 0.625) was not.

## Discussion

Findings from the present study suggest that assessment of cortical function characteristics disclose a spectrum of change between ALS, ALS-Cog and ALS-FTD. Specifically, the advent of cortical hyperexcitability, identified by reduced SICI, was evident across all patient cohorts. However, a reduced RMT, was evident only in ALS-FTD, which suggests a greater level of cortical derangement in ALS coexistent with FTD. While the finding of reduced RMT in itself does not provide conclusive evidence of a greater level of cortical derangement in the overlap phenotypes, evidence from other studies including structural changes in the motor cortex^13^ associated with behavioural and cognitive impairment in ALS; and presence of motor dysfunction being a predictor of shorter survival in the overlap phenotype^53^ lend further support to this idea. Future studies involving measurement of fine motor tasks in ALS subtypes and their relationship with overall cognitive and behavioural impairment would shed further light on cortical derangement and its relationship with disease progression.

Neuropathological evaluation has revealed that TDP-43 pathology appears to propagate from the motor cortex towards frontal areas in ALS^7^ and from frontal areas posteriorly in FTD^40^. Therefore, one possible explanation for the observation of a greater level of cortical excitability could be a greater disease burden in ALS-FTD.

The distinct cortical excitability pattern may also be reflective of different patterns of TDP-43 pathology in the neocortex. In ALS, Betz cells as well as interneuronal involvement have been well recognised^4^. Since the description of TDP-43 as the common pathological link between ALS and FTD^6^, subtypes of TDP-43 have been described, particularly in FTD^41^. Interestingly, type B pathology which involves all cortical layers, seems to be frequent in ALS-FTD overlap while other types involving more superficial cortical layers where interneurons are prominent, may be more common in other FTD phenotypes.

The interpretation of RMT alterations in more complex. Betz cell involvement is an early feature in ALS^4,42^. Prior to the development of TT-TMS techniques, motor cortical neurophysiological descriptions in ALS found reduction in RMT in earlier phases of ALS^43^, which tended to increase with time^44^. While the precise mechanisms underlying this evolution are not well understood, one proposition is that the hyperexcitability may be evidence of an initial disease trigger for the neurodegenerative cascade, followed by progressive cell destruction and therefore an inexcitable motor cortex in later disease stages^2^. In the current study, it is noteworthy that the disease duration at the time of evaluation of cognition, behaviour and motor cortical function, was similar between ALS, ALS-FTD and ALS-Cog. Thus, the observed variation in RMT is more likely to represent underlying disease pathophysiology in the three phenotypes, rather than a representation of disease time course per se.

The physiological basis for SICI generation is thought to represent GABAa receptor mediated inhibition^17,18^. The evidence for this comes from pharmacological studies showing direct (for eg. with lorazepam) and indirect (for eg. using dopamine agonists or antiglutaminergic agents) modulation of the GABAa receptor system leading to increased SICI^18^. The loss of GABAa mediated inhibition may be a key factor in frontal neurodegeneration. This idea is supported by emerging evidence from TMS studies in neurodegenerative dementias^50^ as well as pathological studies demonstrating loss of GABAergic cells in the frontal cortices in FTD brains^51^.

Another interesting observation in the clinical profile is that the ALS-FTD patients were more impaired in the fine motor component of the ALSFRS-R scale, while overall motor impairment was comparable to other groups. Since fine motor function is more likely to represent problems with adaptive complex motor skills, this lends some support for the cortical primacy theory in ALS^1^ and the key role of the corticomotor neuronal system in disease pathogenesis.

At the pathophysiological level, the findings from this study support the ‘dying forward’ hypothesis in ALS via glutaminergic excitotoxic mechanisms^1,3^. Based on findings of a potentially greater level of cortical excitability in ALS-FTD, it is possible that the excitotoxic processes may be more prominent in the overlap syndrome rather than in ALS and ALS-Cog.

In the same context, it is also interesting to note that in ALS-Cog, SICI levels remained significantly low, although not statistically significantly lower than ALS, in the presence of a preserved RMT. Whether this RMT level may present a stage in evolution from hyperexcitability to inexcitability in the wake of progressive pyramidal cell degeneration, is not possible to ascertain from this data. Longitudinal studies evaluating cortical excitability alterations and clinical phenotype evolution may help clarify this.

The study cohort comprised of just over a third of patients with cognitive and behavioural impairment or frank FTD, which is broadly in keeping with previous estimates in ALS^45^. However, the ALS-Cog group appears smaller, which may be a reflection of the application of the revised diagnostic criteria which aim to avoid overestimation of cognitive deficits^29^. Additionally, corrected measures were used in the presence of significant bulbar dysfunction.

Putting our findings in the context of the continuum theory of ALS and FTD^24^, the changes in motor cortical function don’t necessarily imply evolution from ALS-Cog to ALS-FTD. It is possible that the patients who have the overlap syndrome may have a distinct trajectory from the onset of disease, given that the RMT and SICI were both reduced simultaneously. Also, clinical studies in ALS have found that cognitive and behavioural symptoms may precede the onset of motor dysfunction in ALS^46^ making it more likely that the evolution of ALS-Cog may be different to patients who may develop ALS-FTD. Again, longitudinal studies are required to address these important questions.

This findings from the ROC analysis provide a potential diagnostic and prognostic marker based on cortical physiology characteristics, that could help differentiate ALS and ALS-FTD at a comparable time point in the disease trajectory. Application of SICI in this context has been achieved in a large clinical ALS cohort previously^22^. Recent evidence from large clinical cohorts suggests that cognitive and behavioural dysfunction relates to overall prognosis in ALS^47^. Therefore, the findings from this study provide novel markers that could aid prognostication for clinical trials.

This study has few limitations including a relatively small ALS-Cog group which may limit the statistical power to detect differences to the other two groups. However, as indicated above, the overall prevalence of neuropsychological deficits is in keeping with previous estimates. Additionally, the behavioural tool used in the study did not comprehensively assess all domains that may be affected in ALS, such as changes in eating behaviour^48^. The cognitive and behavioural assessments used in the present study were part of the multidisciplinary protocols that were developed at the recruiting centre^34,36^. Other tools such as the Edinburgh cognitive and behavioural screen (ECAS) may be applied in future studies. Reassuringly, the ECAS has been shown to have good convergent validity with the Addenbrooke’s cognitive examination^52^ with the advantage of additionally capturing executive functions. The present study used additional tests of executive function (Trails test and fluency measures).

One further limitation is the lack of a precise numerical estimate of the population screened for inclusion in the study. However, the distribution of cognitive phenotypes is largely in keeping with expected frequency estimates of cognitive impairment in early stage ALS cohorts^45^.

Overall, the current study has documented a distinct motor cortical physiology profile in ALS-FTD. In the setting of clinical trials, the advent of personalised medicine serves to encourage the development of strategies to better determine prognosis. While physiological assessments using TMS themselves may not be practical in all clinical settings, defining clinical correlates of disease mechanisms elucidated through these techniques would help translate prognostic markers to the clinical setting. This is important for care planning and supporting families while optimising overall clinical care. While motor cortical changes do not necessarily imply disease continuum between ALS and ALS-FTD, cortical function characteristics may have utility in prognostication and potentially, predicting the development of cognitive and behavioural dysfunction in ALS.

## References

[1] Eisen A (2017). Cortical influences drive amyotrophic lateral sclerosis. J. Neurol. Neurosurg Psychiatr..

[2] Vucic S (2013). Transcranial magnetic stimulation and amyotrophic lateral sclerosis: pathophysiological insights. J. Neurol. Neurosurg. Psychiatr..

[3] Kiernan MC (2011). Amyotrophic lateral sclerosis. Lancet.

[4] Nihei K, McKee AC, Kowall NW (1993). Patterns of neuronal degeneration in the motor cortex of amyotrophic lateral sclerosis patients. Acta Neuropathol..

[5] Tan RH (2015). TDP-43 proteinopathies: pathological identification of brain regions differentiating clinical phenotypes. Brain.

[6] Neumann M (2006). Ubiquitinated TDP-43 in frontotemporal lobar degeneration and amyotrophic lateral sclerosis. Science.

[7] Brettschneider J (2013). Stages of pTDP-43 pathology in amyotrophic lateral sclerosis. Ann. Neurol..

[8] Tan RH (2017). Distinct TDP-43 inclusion morphologies in frontotemporal lobar degeneration with and without amyotrophic lateral sclerosis. Acta Neuropathol. Commun..

[9] Genç B (2017). Apical dendrite degeneration, a novel cellular pathology for Betz cells in ALS. Sci. Rep..

[10] Jara JH (2017). Evidence for an early innate immune response in the motor cortex of ALS. J. Neuroinflamm..

[11] Agosta F (2016). Structural brain correlates of cognitive and behavioral impairment in MND. Hum. Brain Mapp..

[12] Mohammadi B (2015). Amyotrophic lateral sclerosis affects cortical and subcortical activity underlying motor inhibition and action monitoring. Hum. Brain Mapp..

[13] Mioshi E (2013). Cortical atrophy in ALS is critically associated with neuropsychiatric and cognitive changes. Neurology.

[14] Filippini N (2010). Corpus callosum involvement is a consistent feature of amyotrophic lateral sclerosis. Neurology.

[15] Lillo P (2012). Grey and white matter changes across the amyotrophic lateral sclerosis-frontotemporal dementia continuum. PLoS ONE.

[16] Vucic S, Cheah BC, Kiernan MC (2009). Defining the mechanisms that underlie cortical hyperexcitability in amyotrophic lateral sclerosis. Exp. Neurol..

[17] Vucic S, Kiernan MC (2017). Transcranial magnetic stimulation for the assessment of neurodegenerative disease. Neurotherapeutics.

[18] Vucic S, Kiernan MC (2006). Novel threshold tracking techniques suggest that cortical hyperexcitability is an early feature of motor neuron disease. Brain.

[19] Menon P, Kiernan MC, Vucic S (2015). Cortical hyperexcitability precedes lower motor neuron dysfunction in ALS. Clin. Neurophysiol..

[20] Vucic S, Kiernan MC (2008). Cortical excitability testing distinguishes Kennedy’s disease from amyotrophic lateral sclerosis. Clin. Neurophysiol..

[21] Vucic S, Nicholson GA, Kiernan MC (2010). Cortical excitability in hereditary motor neuronopathy with pyramidal signs: comparison with ALS. J. Neurol. Neurosurg. Psychiatr..

[22] Menon P (2015). Sensitivity and specificity of threshold tracking transcranial magnetic stimulation for diagnosis of amyotrophic lateral sclerosis: a prospective study. Lancet Neurol..

[23] Shibuya K (2016). Motor cortical function determines prognosis in sporadic ALS. Neurology.

[24] Burrell JR (2016). The frontotemporal dementia-motor neuron disease continuum. Lancet Neurol..

[25] Burrell JR, Kiernan MC, Vucic S, Hodges JR (2011). (2011) Motor Neuron dysfunction in frontotemporal dementia. Brain.

[26] Montuschi A (2015). Cognitive correlates in amyotrophic lateral sclerosis: a population-based study in Italy. J. Neurol. Neurosurg. Psychiatr..

[27] Costa J, Swash M, de Carvalho M (2012). Awaji criteria for the diagnosis of amyotrophic lateral sclerosis. Arch. Neurol..

[28] de Carvalho M (2008). Electrodiagnostic criteria for diagnosis of ALS. Clin. Neurophysiol..

[29] Strong MJ (2017). Amyotrophic lateral sclerosis—frontotemporal spectrum disorder (ALS-FTSD): revised diagnostic criteria. Amyotroph. Lateral Scler. Front. Degener..

[30] Turner MR (2004). Evidence of widespread cerebral microglial activation in amyotrophic lateral sclerosis: an [11C](R)-PK11195 positron emission tomography study. Neurobiol. Dis..

[31] Cedarbaum JM (1999). The ALSFRS-R: a revised ALS functional rating scale that incorporates assessments of respiratory function. J. Neurol. Sci..

[32] Kimura F (2006). Progression rate of ALSFRS-R at time of diagnosis predicts survival time in ALS. Neurology.

[33] Labra J (2016). Rate of disease progression: a prognostic biomarker in ALS. J. Neurol. Neurosurg. Psychiatr..

[34] Hsieh S (2013). Validation of the Addenbrook’s cognitive examination III in frontotemporal dementia and Alzheimers disease. Dement Geriatr. Cogn. Disord..

[35] Reitan RM, Wolfson D (1985). The Halstead-Reitan neuropsychological test battery.

[36] Mioshi E (2014). A novel tool to detect behavioural symptoms in ALS. Amyotroph. Lateral Scler. Front. Degener..

[37] Fisher RJ (2002). Two phases of intracortical inhibition revealed by transcranial magnetic threshold tracking. Exp. Brain Res..

[38] Cantello R, Gianelli M, Civardi C, Mutani R (1992). Magnetic brain stimulation: the silent period after the motor evoked potential. Neurology.

[39] Geevasinga N (2016). Riluzole exerts transient modulating effects on cortical and axonal hyperexcitability in ALS. Amyotroph. Lateral Scler. Front. Degener..

[40] Brettschneider J (2014). Sequential distribution of pTDP-43 pathology in behavioral variant frontotemporal dementia (bvFTD). Acta Neuropathol..

[41] Tan RH (2013). Classification of FTLD-TDP cases into pathological subtypes using antibodies against phosphorylated and non-phosphorylated TDP43. Acta Neuropathol. Commun..

[42] Eisen A, Kim S, Pant B (1992). Amyotrophic lateral sclerosis (ALS): a phylogenetic disease of the corticomotoneuron?. Muscle Nerve.

[43] Mills KR, Nithi KA (1997). Corticomotor threshold is reduced in early sporadic amyotrophic lateral sclerosis. Muscle Nerve.

[44] Mills KR (2003). The natural history of central motor abnormalities in amyotrophic lateral sclerosis. Brain.

[45] Phukan J (2012). The syndrome of cognitive impairment in amyotrophic lateral sclerosis: a population-based study. J. Neurol. Neurosurg. Psychiatr..

[46] Mioshi E (2014). Neuropsychiatric changes precede classic motor symptoms in ALS and do not affect survival. Neurology.

[47] Westeneng H-J (2018). Prognosis for patients with amyotrophic lateral sclerosis: development and validation of a personalised prediction model. Lancet Neurol..

[48] Ahmed RM (2016). Amyotrophic lateral sclerosis and frontotemporal dementia: distinct and overlapping changes in eating behaviour and metabolism. Lancet Neurol..

[49] Agarwal S (2018). Primary lateral sclerosis and the amyotrophic lateral sclerosis–frontotemporal dementia spectrum. J. Neurol..

[50] Benussi A (2020). Classification accuracy of transcranial magnetic stimulation for the diagnosis of neurodegenerative dementias. Ann Neurol..

[51] Ferrer I (1999). Neurons and their dendrites in frontotemporal dementia. Dement Geriatr. Cogn. Disord..

[52] De Icaza Valenzuela MM, Bak TH, Pal S, Abrahams S (2018). The Edinburgh cognitive and behavioral ALS screen: relationship to age, education, IQ and the Addenbrooke’s cognitive examination-III. Amyotroph. Lateral Scler. Front. Degener..

[53] Ahmed RM (2020). Phenotypic variability in ALS-FTD and effect on survival. Neurology.

[54] Huynh W (2016). Assessment of the upper motor neuron in amyotrophic lateral sclerosis. Clin. Neurophysiol..

[55] Agarwal S (2019). Interrogating cortical function with transcranial magnetic stimulation: insights from neurodegenerative disease and stroke. J. Neurol. Neurosurg. Psychiatr..

[56] Shefner JM (2020). A proposal for new diagnostic criteria for ALS. Clin. Neurophysiol..

